# Evidence of secondary anopheline vectors in sustaining malaria transmission in Kokrajhar District, Assam, Northeastern India

**DOI:** 10.1186/s13071-025-07110-5

**Published:** 2025-11-21

**Authors:** Kuldeep Singh, Ajeet Mohanty, Waseem Akram Malla, Ritesh Ranjha, Jugal Gam, Rahim Ali, Praveen Kumar Bharti, Anup R. Anvikar, Himmat Singh

**Affiliations:** 1 ICMR-National Institute of Malaria Research, Dwarka Sector 8, New Delhi, 110077 India; 2 Faculty of Biological Sciences, Academy of Scientific and Innovative Research (AcSIR), 11, Ghaziabad, Uttar Pradesh India 201002; 3 ICMR-National Institute of Malaria Research Field Station, Goa, 781022 India; 4 ICMR-National Institute of Malaria Research Field Station, Guwahati, Assam 781022 India; 5 State Vector Borne Disease Control Programme, Kokrajhar, Assam India; 6 State Vector Borne Disease Control Programme, Baksa, Assam India

**Keywords:** *Anopheles maculatus*, Secondary vector, *Plasmodium vivax*, *Plasmodium falciparum*

## Abstract

**Background:**

In the northeastern region of India, perennial malaria transmission persists in certain hotspots in areas geographically adjacent to the international borders with Bhutan, Bangladesh, and Myanmar, where both *Plasmodium vivax* and *Plasmodium falciparum* coexist, particularly in remote, forested, and inaccessible areas. This northeastern landscape harbors a wide diversity of anopheline vector species; *Anopheles minimus* and *Anopheles baimaii* are the traditional primary vectors of malaria. The extensive deployment of long-lasting insecticidal nets (LLINs) and indoor residual spraying as traditional vector control strategies has resulted in regional and temporal changes in species composition, specifically *An. minimus* and *An. baimaii*, and their resting and feeding behavior. Despite the reduced abundance of these primary anopheline vectors, the persistence of malaria suggests the involvement of additional anopheline species. Secondary malaria vectors may also play a role in transmitting malaria, along with primary malaria vectors, and are widely distributed across northeastern India. Secondary malaria vectors have significantly lower sporozoite rates compared with primary malaria vectors, yet are capable of sustaining malaria transmission in a specific region. This study aimed to investigate the sporozoite positivity of secondary anopheline species in the high-malaria-endemic district of Kokrajhar, Assam, in northeastern India.

**Methods:**

During the study period, 1794 female mosquitoes representing five genera in *Anopheles*, *Culex*, *Aedes*, *Mansonia*, and *Armigeres* were collected using three methods: CDC light trap collection, indoor resting collection using the mouth aspiration method, and pyrethrum spray captures. Morphologically identified *Anopheles maculatus* group specimens were validated by polymerase chain reaction targeting the Internal Transcribed Spacer 2 region within the nuclear ribosomal DNA and referred to as *An. maculatus,* a species of the Maculatus Group of subgenus Cellia (Diptera: Culicidae)*.*

**Results:**

The *Plasmodium* positivity (Percent, number/total number) was highest in *An. maculatus* (4%; 5/80), followed by *An. minimus* (4.8%; 1/21), and *Anopheles kochi* (4.6%; 1/22)*.* These results suggest that anopheline species beyond the traditionally recognized primary vectors, such as *An. minimus* and *An. baimaii*, may play a role in sustaining malaria transmission in endemic areas of northeastern India.

**Conclusions:**

Recognizing and integrating the behavior and ecology of secondary vectors into malaria control programs is essential for the development and deployment of more targeted and sustainable vector control strategies.

**Graphical abstract:**

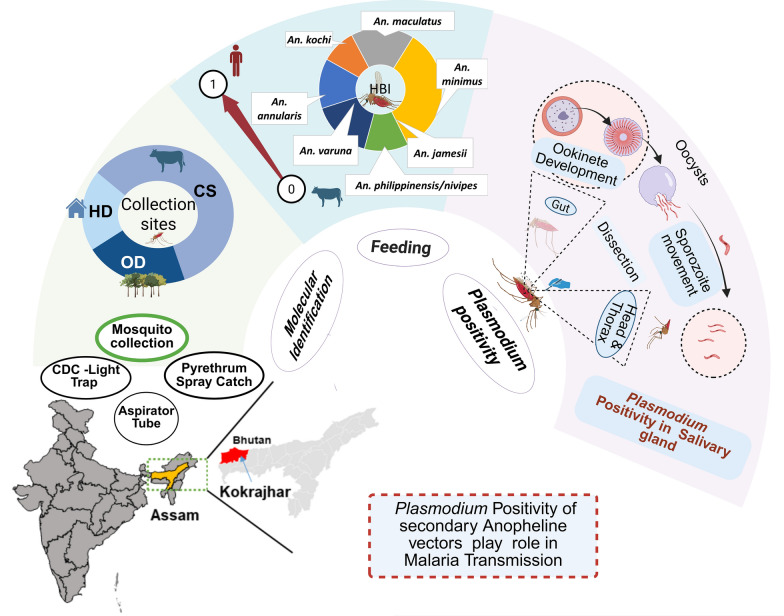

**Supplementary Information:**

The online version contains supplementary material available at 10.1186/s13071-025-07110-5.

## Background

Southeast Asian countries harbor a wide diversity of anopheline vector species that transmit malaria throughout tropical and subtropical foci, leading to perennial transmission [1–4]. These *Anopheles* species exhibit variable transmission capabilities and may function as primary vectors in one geographical area while being classified as secondary or non-vectors in other malaria-endemic regions [5, 6]. The widespread implementation of long-lasting insecticidal nets (LLINs) and indoor residual spraying as core vector control measures has led to a spatial and temporal alteration in the species composition, vector densities near human dwellings, and feeding behavior of the mosquitoes [7, 8]. Furthermore, deforestation and climate change have likely facilitated the spread and re-establishment of secondary anopheline species in the region. The secondary vectors, such as *Anopheles aconitus, Anopheles annularis, Anopheles jeyporiensis, Anopheles maculatus*, and *Anopheles philippinensis*, which are typically zoophilic and exophilic, have historically received limited attention in transmission studies [9]. Nevertheless, their potential contribution to malaria transmission should not be underestimated, especially given their high population densities, occasional endophily, and confirmed positivity for *Plasmodium* sporozoites comparable to that of primary vectors, such as *An. minimus* and *An. baimaii* [10, 11]*.* A recent study by Zang et al. (2022) reported high *Plasmodium* positivity in eight anopheline species, including *Anopheles sinensis, An. kochi, Anopheles. vagus, Anopheles minimus, An. annularis, Anopheles philippinensis, Anopheles tessellatus,* and *Anopheles dirus* [12]. All the above species were found to be positive for *Plasmodium. vivax*, except *An. dirus*, which was positive for *Plasmodium falciparum*, and *An. tessellatus*, which manifested with the co-infection of *P. vivax* and *P. falciparum*. Similarly, *An. kochi* has also been found to be *Plasmodium* positive from Laos, Cambodia, and Indonesia [13, 14], *An. vagus* positive from Indonesia [14], Cambodia [15], and Bangladesh [16], while *An. karwari* is considered a secondary vector in the Australian region [17]. *Anopheles maculatus* is regarded as a major vector in rural and forested areas of West Malaysia, as well as in regions of Sumatra and Java, Indonesia [5, 18]. In the Indian subcontinent, *An. baimaii* (formerly dirus D), *An. minimus, An. sundaicus, An. philippinensis, An. aconitus, An. annularis,* and *An. vagus* have shown *Plasmodium* positivity in Bangladesh up to the 1990s [16]. In 2009–2010, *An. karwari*, *An. maculatus, An. barbirostris, An. nigerrimus* and *An. subpictus* were also shown to carry the *Plasmodium* sporozoite in Bangladesh [16, 17]. In India, the majority of malaria cases are attributed to six primary vectors: *Anopheles culicifacies*, *Anopheles fluviatilis*, *An. minimus, Anopheles stephensi*, *Anopheles baimaii*, and *Anopheles sundaicus*. However, secondary vectors, such as *An. annularis*, *An. philippinensis/nivipes*, and *An. varuna*, also significantly contributes to malaria transmission and is widely distributed throughout the malaria-endemic regions. [19–21]. In the northeastern states of India. *Plasmodium* positivity in several other *Anopheles* species, including *An. vagus* [20], *An. aconitus* [22], *An. hyrcanus* group [22], *An. jeyporiensis* [22], *An. kochi* [22], and *An. tessellatus* [22] has been reported from the northeastern states of India.

Data on the species composition, spatial distribution, and vectorial capacity of the *Maculatus* group in India remains limited, hindering a comprehensive understanding of the evolving malaria epidemiology in the region. In the northeastern region of India, six-member species of the *Maculatus* group: *An. maculatus, An. pseudowillmori, An. sawadwongporni, An. dravidicus, An. willmori*, and *An. rampae* were prevalent species across the majority of northeastern India, with *Anopheles willmori* exhibiting a limited distribution confined to Nagaland. Additionally, *An. sawadwongporni* from Meghalaya, Mizoram, and *An. rampae* was noted explicitly in Mizoram state [23–25]. However, these species from the *Maculatus* group were not reported to be positive for sporozoites by Singh et al. (2012). Malaria transmission in the northeastern states of India, particularly along the international borders, poses a significant public health challenge [26]. The geographical location and persistent malaria transmission in the Kokrajhar District of Assam (India) underscore the need to understand the role of secondary vectors in regional malaria transmission dynamics, especially over the dry season. The present study seeks to detect *Plasmodium* sporozoite positivity and analyze the host preference of secondary vectors during the dry season, when some anopheline species remain active. Identifying these vectors helps highlight the potential for residual malaria transmission, which is often overlooked when focusing solely on high transmission seasons.

## Methods

### Study site

**Fig. 1:**
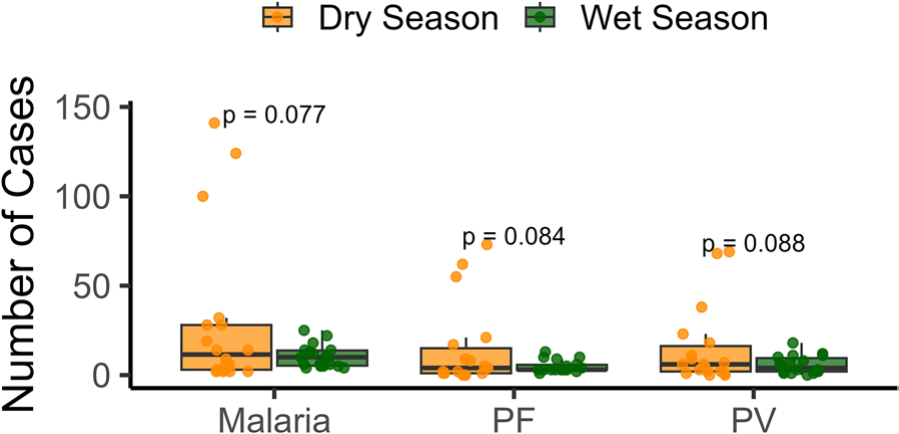
Distribution of reported cases of Malaria, *Plasmodium falciparum* (*Pf*), and *Plasmodium vivax* (*Pv*) during the dry season (orange) and wet season (green). Each box represents the interquartile range (IQR), with the horizontal line indicating the median number of cases

The study was conducted at the Kachugaon Block Primary Health Center (PHC) in Kokrajhar district, Assam, India, situated along the foothills bordering Bhutan, characterized by deep forests and slow-moving streams throughout the year. Kokrajhar district, part of the Bodoland Territorial Region, is one of the five districts in the state endemic to malaria. Its strategic location bordering Bhutan to the north and West Bengal to the west, along with its unique ecological and socio-demographic features, makes it a persistent malaria hotspot [27]. The region’s tropical climate, high rainfall, forested terrain, moderate temperature range of 22–32 °C, and extensive rural areas provide favorable breeding conditions for multiple *Anopheles* species, including *An. minimus, An. annularis, An. culicifacies, An. vagus*, and *An. maculatus* [26]. The entire geographical area of the Kachugaon Block Primary Health Centre (PHC) is within a forested region, with portions of the Dotma and Balajan Block PHCs also located in forest areas [28–30]. Historical malaria data for the district were collected from the state vector-borne diseases control programme for analysis. During the period from 2001 to 2010, the annual parasite index (API) of Kachugaon PHC reached a value of 6.43 in 2003, peaking at 20.71 in 2006. In contrast, the API of Kokrajhar district was 6.08 and 6.55 in the respective years. Over the past two decades, the state of Assam has witnessed a substantial decline in malaria prevalence. In the Kokrajhar district, malaria transmission can be perennial but declining with significant inter-seasonal variation (*p* < 0.001). A notable post-monsoon surge in *P. vivax* cases is observed, with a secondary transmission peak occurring during November and December. During the 2020 dry season, a higher prevalence of malaria was observed, accompanied by an increased number of *P. vivax* cases compared with previous dry seasons. Despite this, seasonal rainfall patterns driven by pre-monsoon activity and the southwest monsoon, which bring an average of 2–3 m of rainfall between April and September, continue to influence transmission dynamics. Figure 1 illustrates the boxplot comparing the number of malaria cases and species-wise (*P. falciparum* and *P. vivax*), between the dry season and wet season. The dry season shows a higher median number of malaria cases compared with the wet season. Although the *p*-values exceed the conventional threshold of 0.05, a noticeable trend is observed, with more cases occurring in the dry season for both *Plasmodium* species.

### Mosquito collection

Entomological collections were conducted using three methods: CDC light traps, pyrethrum spray captures (PSCs), and hand collections with a mouth aspirator and flashlight. CDC light traps were used for mosquito collection from indoor, outdoor, and cattle sheds from dawn to dusk, from 18:00 to 06:00 h. For indoor collection, traps were hung at a fixed height of 1.5 m above the floor, positioned at the foot end of a human-occupied bed. Outdoor and cattle shed CDC light trap collections were made from locations where people assemble in the evening, outside the house, within the peri-domestic space, and cattle sheds, respectively. It was ensured that the Indoor and outdoor sampling houses did not overlap. Anopheline vector density from the CDC light trap collections was presented as the number of female mosquitoes collected per trap night from HD, OD, or CS. Each of the three traps from each location was done monthly.

For daytime resting collections, four fixed and two randomly selected human dwellings (HD), outdoor (OD), and cattle sheds (CS) were selected. The collections were made using flashlights and mouth aspirators during the early morning hours (6:00–8:00) for 15 min at each location. The same entomological team was deployed to collect mosquitoes in the study villages. From the number of female mosquitoes, the per man-hour density (MHD) of each vector species was calculated as the number of mosquitoes collected by one insect collector in 1 hour. Another method used in the study to collect adult mosquitoes resting indoors was the use of PSCs, which were collected once a month. Most, if not all, of the *Anopheles* mosquitoes resting indoors were collected using this method during the morning hours (8:00–10:00 h). In this method, the entire floor of the room (human dwelling) was covered with a white cotton sheet. Using a flit sprayer, the complete room was sprayed with 0.1–0.2% pyrethrum spray, causing all mosquitoes resting inside the room to be knocked down onto the sheet. The collected mosquitoes were then transferred into petri dishes lined with wet cotton or filter paper and transported to the laboratory for further processing. This method provided the total number of mosquitoes and species resting per structure. Mosquitoes collected from different localities were kept in separate test tubes, labelled with location, village name, date, and time of collection, and then brought to the field laboratory for identification and further processing, including host preference and vector incrimination.

### Mosquito species identification and sample preparation

Adult *Anopheles* mosquitoes collected using the light trap and hand catch methods were morphologically identified using standard taxonomic keys [31]. The mosquitoes collected using PSCs were placed on petri plates lined with wet filter paper and transported to the laboratory for further processing. After morphological identification of the species collected by all three methods, each anopheline mosquito was stored in cryovials using silica gel as a mosquito absorbent to prevent microbial growth, labeled with the collection date, collection method, site, morphological identification number, and species.

### Extraction of mosquito genomic DNA and molecular identification

Individual mosquito specimens collected using three methods were dissected into two parts: the head/thorax and the abdomen. Genomic DNA was separately extracted from the thorax/head and abdomen using the QIAamp DNA mini kit (Qiagen, Germany). The isolated DNA was then stored at − 20 °C until further analysis. Morphologically identified *An. maculatus* were further confirmed using the molecular method reported in the literature. A brief description of the primers is provided in Additional File 1: Table S1.

### Detection of *Plasmodium* species using PCR

Each mosquito specimen’s head-thorax and abdomen were screened to detect sporozoites and oocysts, respectively, using the 18S rRNA gene. The rPLU5 and rPLU6 were employed as genus-specific primers for the first step of PCR, whilst rPF1/2 and rPV1/2 functioned as species-specific primers for the detection of *P. falciparum* and *P. vivax* in the second step of PCR, respectively [32–34]. The details of the PCR primers are provided in Additional File 1: Table S1. One negative control, i.e., head-thorax homogenate of laboratory-reared unfed female *An. culicifacies* mosquitoes were prepared identically to the test sample, and one *Plasmodium* genus-positive control was set in each experiment. The PCR products were detected by 2% agarose gel electrophoresis and visualized under ultraviolet transillumination.

### Blood meal analysis

To investigate the anthropophagic behavior of *Anopheles* mosquitoes, the abdomens of all fully fed specimens were analyzed for human blood using PCR, as reported in the literature elsewhere. The specific primers derived from the ribosomal RNA intergenic spacer sequence of *Homo sapiens* and the mitochondrial DNA (mtDNA) of *Bos taurus* are presented in Additional File 1: Table S1. The PCR conditions were followed as previously described [34].

### Data analysis

Data collected during the study period were entered into Microsoft Excel 2021 version for cleaning and analyzed using SPSS, leveraging Excel’s basic functionalities and SPSS’s advanced statistical capabilities. For the light trap collection, findings are reported as the number of individuals per trap per night. The per Man-Hour Density (MHD) determination was based on the total number of mosquitoes (N) collected, the time spent in minutes (T), and the number of insect collectors involved in the collection (C). The formula for MHD is: MHD = N × (60/T) × C. The PSCs were reported as the number of mosquitoes collected per structure. The human blood index was determined by dividing the number of Anopheles mosquitoes fed on humans by the total number of fully fed mosquitoes. The sporozoite positivity was reported as a percentage of specific *Anopheles* species. The difference was considered statistically significant when the *P* value was less than 0.05.

## Results

### Anopheline species relative composition

**Fig. 2:**
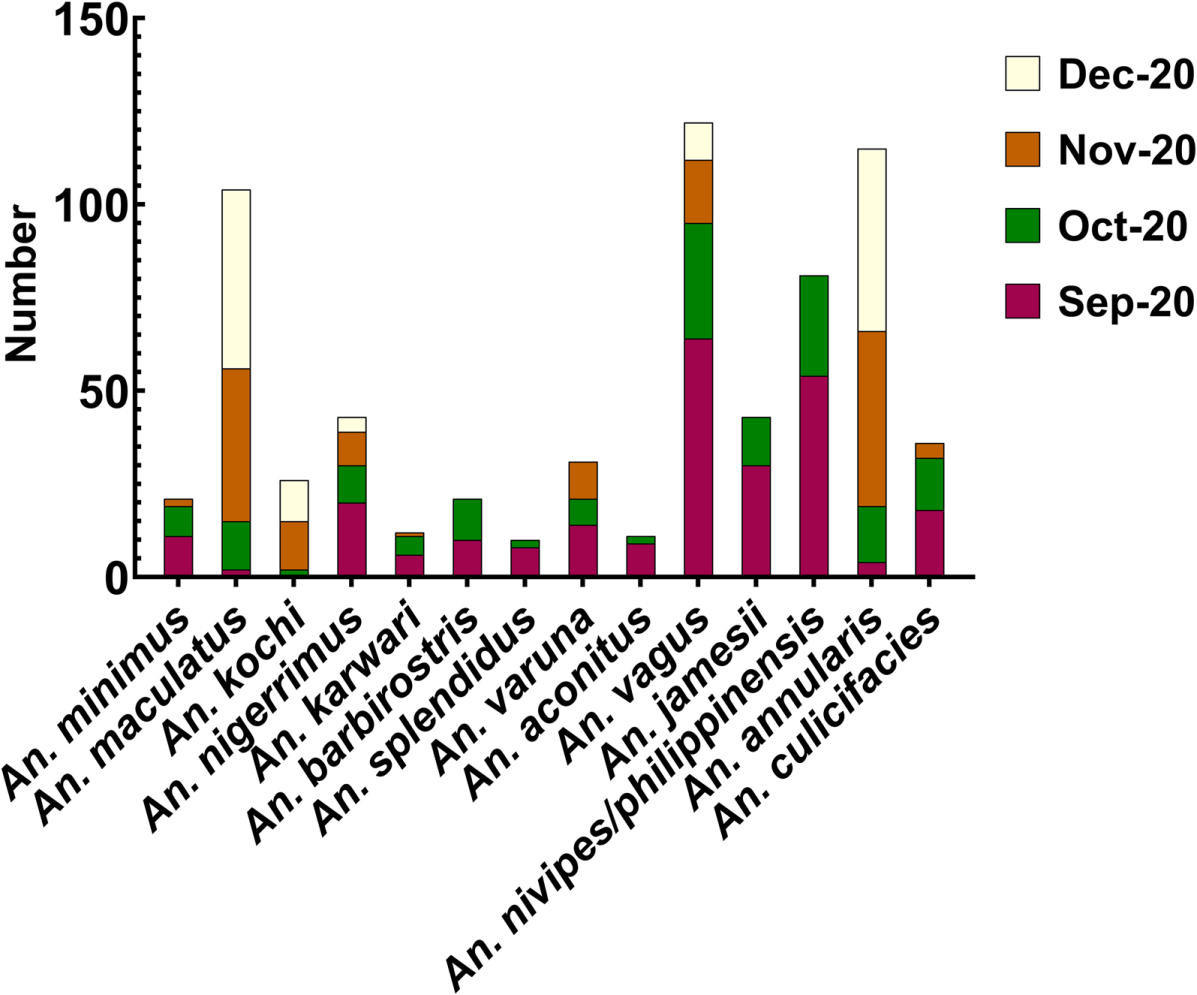
Monthly variation in Anopheles species abundance (Sep–Dec 2020), collected from human dwellings, outdoors, and cattle sheds using different methods (CDC light traps, day-resting collections with mouth aspiration, and pyrethrum spray captures)

**Fig. 3:**
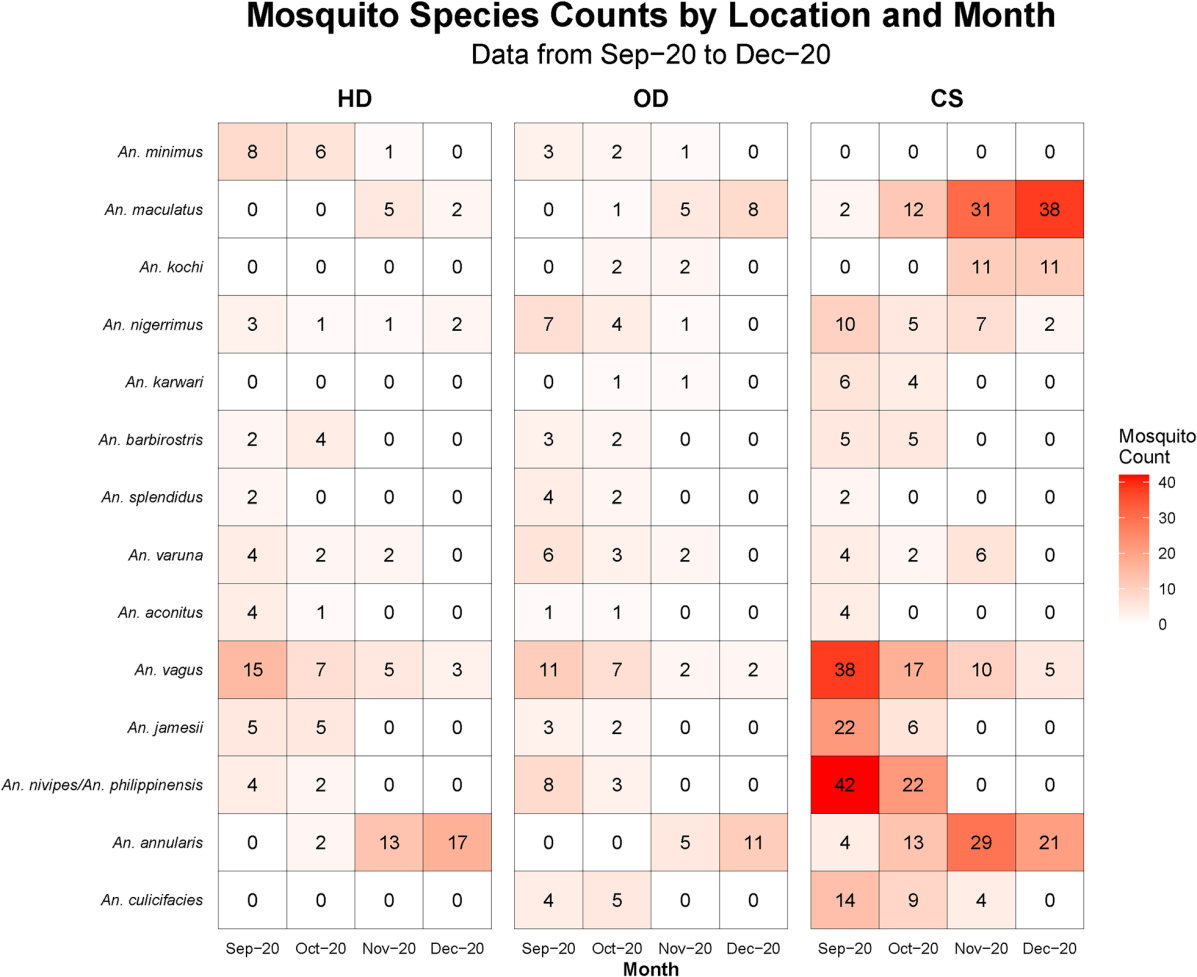
Heat plot showing the absolute mosquito densities across different months and habitats (human dwellings, cattle sheds, and outdoor sites). Human dwellings are housing structures where people reside. Outdoor and cattle sheds are the locations where people assemble in the evening, outside the house, within the peri-domestic space, and cattle sheds, respectively

During the study period from September 2020 to December 2020, a total of 1794 female mosquitoes belonging to five genera in *Culex, Anopheles, Aedes, Mansonia, and Armigeres* were collected. Among these, *Culex* was the predominant genus, comprising 61.1%, followed by *Anopheles* (37.68%). Figure 2 depicts the different anopheline species collected from September to December 2020 from human dwellings, outdoors, and cattle sheds using different methods (CDC light traps, day-resting collections with mouth aspiration, and pyrethrum spray captures). From September to October 2020, *An. vagus* and *An. nivipes/philippinensis* were dominant, yet *An. maculatus* and *An. annularis* were predominant in later months. Six hundred seventy-six female *Anopheles* mosquitoes representing 14 species were systematically collected from the Kachugaon PHC, Kokrajhar District, Assam, India. Morphologically identified *Anopheles maculatus* group specimens collected from all three methods were validated by polymerase chain reaction targeting the ITS-2 region within the nuclear ribosomal DNA and referred to as *An. maculatus*, a species of the Maculatus Group of subgenus Cellia (Diptera: Culicidae), throughout the manuscript. A total number (*n*) of 414 anopheline mosquitoes were collected using the light trap method, followed by the hand catch method (*n* = 241), while the spray catch method yielded the lowest with 21 specimens. Notably, *An. varuna, An. aconitus, An. vagus* and *An. minimus* were collected using the spray catch method in human dwellings. The most prevalent mosquito species was *An. vagus* (18.7%)*, An. annularis* (17.6%), *An. maculatus* (16.0%), and *An. philippines/nivipes* (12.4%). *An. minimus* was 3.2%. Importantly, *An. baimaii* was not observed during the study period. Figure 3 illustrates the faceted heatmap, which shows the relative abundance of different *Anopheline* species across the study months and collection sites. The heat intensity exhibits seasonal variation, with a higher abundance during the post-monsoon months (September–October) and a decline toward December. Species composition also varied by habitat, with cattle sheds, outdoor, and human dwellings showing distinct patterns. A significant difference was observed in the abundance of anopheline species collected from HD and CS (Kruskal–Wallis *H *test, *H* = 6.03, *df* = 2, *P* < 0.041), with a mean rank score of 15.4 for HD and 30.41 for CS, indicating higher species occurrence in CS. Interestingly, no *An. minimus* was found in the CS. Similarly, a significant difference was observed in the abundance of anopheline species from September 2020 to December 2020 (Kruskal–Wallis *H* test, *H* = 2.65, *df* = 3, *P* < 0.041), with mean rank scores of 38.83 for September 2020, 37.28 for October 2020, 29.78 for November 2020, and 40.11 for December 2020.

### *Plasmodium* positivity

The *Plasmodium* infection rate in *An. maculatus* was 5.0%, followed by *An. minimus* 4.8%, and 4.6% in *An. kochi.* There was no sporozoite positivity in *An. culicifacies ss* and *An. jamseii* during the study*.* All *Plasmodium*-positive samples were identified as *P. vivax* (Additional File 2: Fig. S1). PCR amplification of the thoracic region of *An. maculatus* and *An. kochi* revealed a genus-specific band at 1100 bp for the *Plasmodium* genus*,* and the presence of 120 bp confirmed the detection of *P. vivax*. The detailed description of % *Plasmodium* positivity is shown in Table 1.

**Table 1:** Summary information on detecting Human *Plasmodium* species and anthropophagic behavior in Anopheles species

*Anopheles* species	No. collected	No. tested	*Plasmodium* sp. (number)		*Plasmodium* sp. (%)		Human blood index		
			Abdomen	Thorax	Abdomen	Thorax	No. tested	Positive human blood	HBI_a_
*An. annularis*	108	43	0	1	0	2.3	21	7	0.33
*Anopheles kochi*	22	22	0	1	0	4.6	13	3	0.23
*An. maculatus*	82	80	1	4	1.3	5.0	19	8	0.42
*An. minimus*	21	21	0	1	0	4.8	7	6	0.86
*An. jamesii*	30	19	0	0	0	0.0	25	0	0.00
*An. philippinensis/nivipes*	57	41	0	0	0	0.0	17	5	0.29
*An. varuna*	26	25	0	0	0	0.0	5	2	0.40

_**a**_HBI: human blood index

### Blood meal analysis in anopheline species

The mean HBI for all full-fed anopheline mosquitoes was 0.36 (95% confidence interval: 0.60–0.02, *p* < 0.036). The highest HBI of 0.86 was observed for *An. minimus* followed by *An. maculatus,* which was 0.42. However, *An. jamseii* was not found positive for human blood (Table 1).

## Discussion

India has made substantial progress in reducing malaria incidence over the past decade. Key vector control strategies, including the widespread distribution of LLINs and IRS, have significantly contributed to the decline in malaria cases and a reduction in the population density of primary malaria vectors [35–37]. This study has investigated the sporozoite positivity and blood meal sources of anopheline species in malaria-endemic Kachugaon PHC of Kokrajhar, Assam, India. *Anopheles minimu*s and *An. baimaii* are well-established as the principal malaria vectors in the northeastern states of India. In addition to these primary vectors, other anopheline species, including *An. aconitus*, *An. annularis sl., An. nivipes/philippinensis, An. kochi*, *An. culicifacies*, and *An. vagus* have been reported to carry variable levels of circumsporozoite protein positivity and anthropophagy in the foothills regions of Assam [38–40]. Singh et al. (2012) tested 351 *An. maculatus* group mosquitoes across all eight northeastern states of India, but no natural *Plasmodium* infection was detected [23]. However, *P. vivax* CSP positivity in *An. maculatus* was reported from the Habiganj district in Bangladesh, a region bordering India, indicating possible cross-border transmission dynamics [41]. Over the past decade, malaria epidemiology in the forested areas of Kokrajhar district has demonstrated fluctuating trends in *P. falciparum* and *P. vivax* case prevalence, with an increasing number of *P. vivax* cases, particularly during the dry season. In the present investigation, *An. minimus* was found to a lesser extent, and *An. baimaii* was not detected in the study villages. The abundance of *An. minimus* was only present in human dwellings and *An. minimus* were collected from the sampled cattle sheds. The sporozoite positivity in *An. maculatus*, *An. kochi*, *An. annularis* and *An. minimus* revealed a role of multiple anopheline species in malaria transmission. Temporal changes in anopheline species composition from September to December 2020, along with sporozoite positivity in previously under-recognized vectors, may be linked to the persistent, perennial malaria burden in the Kokrajhar district of Assam, India. These findings also indicate that anopheline species other than *An. minimus* and *An. baimaii* might have a role in malaria transmission in endemic areas of northeastern India. However, further in-depth spatial and temporal studies focusing on the bionomics of these lesser-known vectors are required before they can be firmly established as key contributors to malaria transmission. The results of this study are consistent with existing data from neighboring countries and other regions with similar ecological and epidemiological contexts.

The study was conducted over a brief period in a high malaria-endemic PHC of Assam, selected due to the unexpectedly high malaria transmission observed during the dry season of the previous year, despite a notably low density of primary malaria vectors. However, mosquito sampling was constrained by the small number of mosquitoes collected and the lack of year-round sampling. The small sample size of some anopheline species may lead to insufficient statistical power. A larger sample size with longitudinal sampling across multiple years would help generate evidence for residual malaria transmission. Further studies are required to gain an in-depth understanding of the vector bionomics and residual transmission in the region, which is strategically located along the international border with Bhutan.

## Conclusions

The study highlights the variability in the density of primary malaria vectors and the detection of sporozoite positivity in other anopheline species during the post-rainy season in the malaria-endemic region of Kokrajhar district, Assam, India. This study provides evidence that *Anopheles* species traditionally considered secondary vectors may substantially contribute to malaria transmission in Kokrajhar, Assam. The detection of *Plasmodium* circumsporozoite proteins in various anopheline species, combined with their high densities, exophilic and zoophilic behaviors, highlights their potential role in sustaining residual and outdoor malaria transmission. These findings underscore the importance of expanded entomological surveillance and targeted vector control strategies in better understanding the bionomics and transmission potential of these understudied *Anopheles* vectors.

## Supplementary Information

Supplementary Materal 1.Table S1. Details of primers for the identification of *Anopheles maculatus*, the detection of human blood, bovine blood, and *Plasmodium* parasites.

Supplementary Material 2.Fig. S1. PCR detection of *Plasmodium* DNA in the thorax region of *Anopheline* species: Panel A shows detection of *Plasmodium* DNA- Lane 1: (100 bp DNA), Lane 2: Positive control, Lane 3: -ve control, Lane 4: *Anopheles maculatus,* Lane 5: *Anopheles kochi* showing band at ~ 1100 bp; Panel B shows confirmation via *Plasmodium vivax*-specific amplification at band 120 bp-Lane 1: 100 bp ladder, Lane 2: Positive control, Lane 3: Negative control, Lane 4: *Anopheles maculatus*; Lane 5: *Anopheles kochi* showing amplification at 120 bp.

## Data Availability

Data supporting the main conclusions of this study are included in the manuscript.

## Abbreviations

LLINs: Long-lasting insecticidal nets
PSCs: Pyrethrum spray captures
IRS: Indoor Residual Spray
PHC: Primary Health Centre
API: Annual Parasite Index
SFR: Slide Falciparum Rate
CS: Cattle Shed
HD: Human Dwelling
OD: Outdoors
HBI: Human Blood Index
