# Supplementary figures and images for: Evidence of secondary anopheline vectors in sustaining malaria transmission in Kokrajhar District, Assam, Northeastern India
Source: Parasit Vectors. 2025 Nov 21;18:476. doi: 10.1186/s13071-025-07110-5 (PMC12639997; doi:10.1186/s13071-025-07110-5)

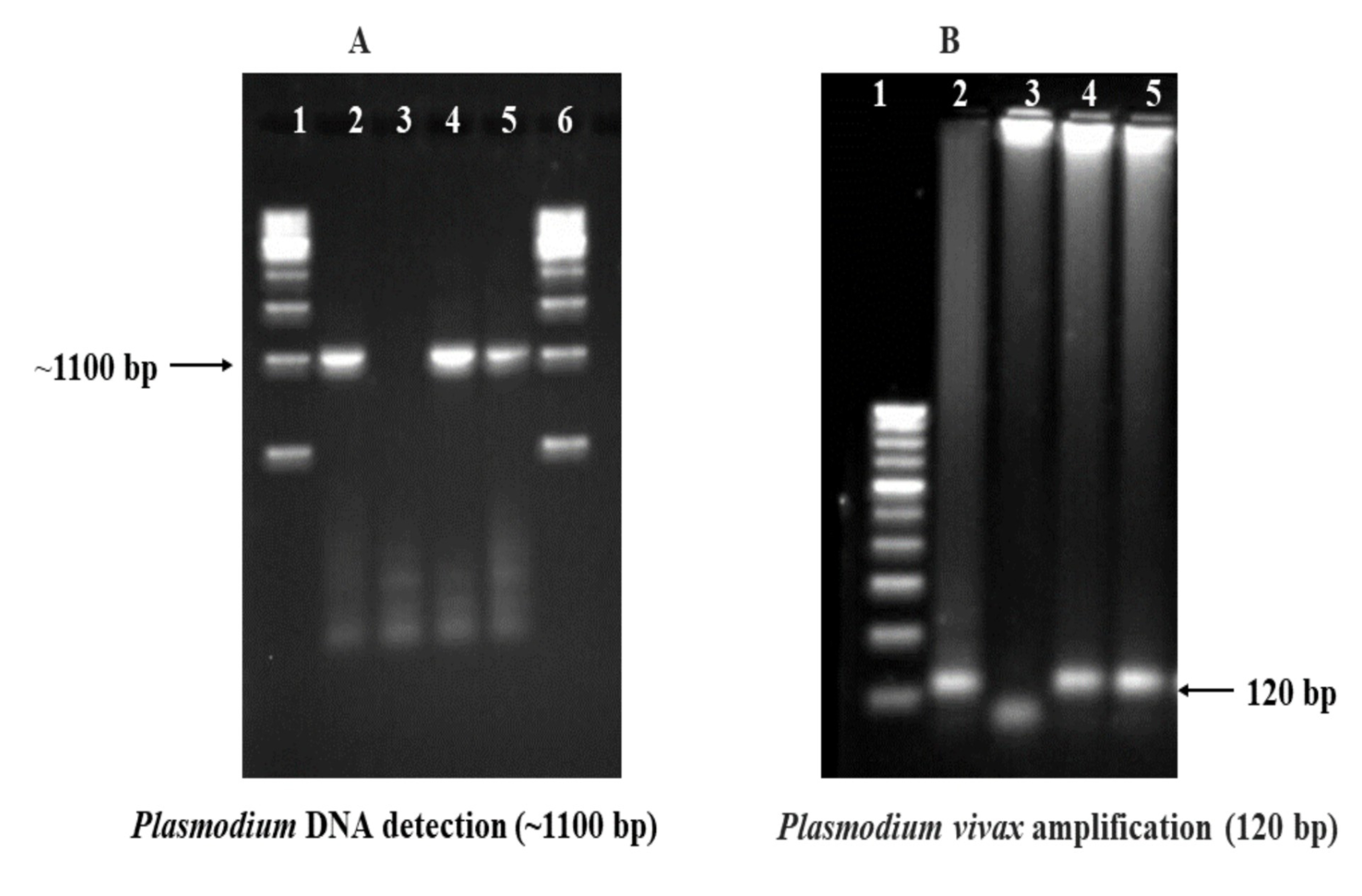

Supplement: Supplementary file 1 — Supplementary Materal 1.Table S1. Details of primers for the identification of Anopheles maculatus, the detection of human blood, bovine blood, and Plasmodium parasites. [file 13071_2025_7110_MOESM1_ESM.jpg]
