# Supplementary material for: Evidence of secondary anopheline vectors in sustaining malaria transmission in Kokrajhar District, Assam, Northeastern India
Source: Parasit Vectors. 2025 Nov 21;18:476. doi: 10.1186/s13071-025-07110-5 (PMC12639997; doi:10.1186/s13071-025-07110-5)
Supplement: Supplementary file 2 — Supplementary Material 2.Fig. S1. PCR detection of Plasmodium DNA in the thorax region of Anopheline species: Panel A shows detection of Plasmodium DNA- Lane 1: (100 bp DNA), Lane 2: Positive control, Lane 3: -ve control, Lane 4: Anopheles maculatus, Lane 5: Anopheles kochi showing band at ~ 1100 bp; Panel B shows confirmation via Plasmodium vivax-specific amplification at band 120 bp-Lane 1: 100 bp ladder, Lane 2: Positive control, Lane 3: Negative control, Lane 4: Anopheles maculatus; Lane 5: Anopheles kochi showing amplification at 120 bp. [file 13071_2025_7110_MOESM2_ESM.docx]

**Additional file 1:**

**Table S1 Details of primers for identification of *Anopheles maculatus*, detection of human blood, bovine blood, and *Plasmodium* parasites**

| **Species** | **Primer name** | **Sequence (5′–3′)** | **PCR product size (bp)** | **Target gene** | **Reference** |
| --- | --- | --- | --- | --- | --- |
| *An. maculatus* | ITS2A | TGT GAA CTG CAG GAC A | 460 | ITS2 | [1] |
|  | ITS2B | TAT GCT TAA ATT CAG GGG GT |  |  |  |
| *Homo sapiens* (human) | HUM1 | CGAGAGTTC//TCTGGAAGAATTGA | 519 | rDNA | [2] |
|  | HUM2 | TGATAGCCTGGAAGTGACAAAAT |  |  |  |
| *Bovine Bos taurus* | B1 | CATCATAGCAATTGCCATAGTCC | 165 | mtDNA | [3] |
|  | B2 | GTACTAGTAGTATTAGAGCTAGAATTAG |  |  |  |
| *Plasmodium (*genus-wide) | rPLU5 | CCTGTTGTTGCCTTAAACTTC | 1100 | 18S rRNA | [4] |
|  | rPLU6 | TTAAAATTGTTGCAGTTAAAACG |  |  |  |
| *Plasmodium falciparum* (Species) | rFAL 1 | TTAAACTGGTTTGGGAAAACCAAATATATT  ACACAATGAACTCAATCATGACTACCCGTC | 205 | 18S rRNA | [4] |
| *Plasmodium vivax (Species)* | rVIV1 | CGC T TCTAGCTTAATCCACATAACTGATAC | 120 | 18S rRNA | [4] |
|  | rVIV2 | ACTTCCAAGCCGAAGCAAAGAAAGTCCTTA |  |  |  |

References:

[1] Sum JS, Lee WC, Amir A, Braima KA, Jeffery J, Abdul-Aziz NM, Fong MY, Lau YL. Phylogenetic study of six species of Anopheles mosquitoes in Peninsular Malaysia based on inter-transcribed spacer region 2 (ITS2) of ribosomal DNA. Parasites & vectors. 2014 Dec;7:1-8.

[2] Mohanty A, Kar P, Mishra K, Singh DV, Mohapatra N, Kar SK, et al. Multiplex PCR assay for the detection of *Anopheles fluviatilis* species complex, human host preference, and Plasmodium falciparum sporozoite presence, using a unique mosquito processing method. Am J Trop Med Hyg. 2007;76:837–43.

[3] Corona B, Lleonard R, Carpio Y, Uffo O, Martínez S. PCR detection of DNA of bovine, ovine -caprine and porcine origin in feed as part of a bovine spongiform encephalopathy control program. Spanish J Agri Res. 2007;5:312–7.

[3] Corona B, Lleonard R, Carpio Y, Uffo O, Martínez S. PCR detection of DNA of bovine, ovine -caprine and porcine origin in feed as part of a bovine spongiform encephalopathy control program. Spanish J Agri Res. 2007;5:312–7.

[4] Snounou G, Pinheiro L, Goncalves A, Fonseca L, Dias F, Brown KN, et al. The importance of sensitive detection of malaria parasites in the human and insect hosts in epidemiological studies, as shown by the analysis of field samples from Guinea Bissau. Trans R Soc Trop Med Hyg. 1993;87:649–53.
